# Prodigiosin inhibits bacterial growth and virulence factors as a potential physiological response to interspecies competition

**DOI:** 10.1371/journal.pone.0253445

**Published:** 2021-06-23

**Authors:** Chee-Hoo Yip, Sobina Mahalingam, Kiew-Lian Wan, Sheila Nathan

**Affiliations:** Department of Biological Sciences and Biotechnology, Faculty of Science and Technology, Universiti Kebangsaan Malaysia, Selangor, Malaysia; Nitte University, INDIA

## Abstract

Prodigiosin, a red linear tripyrrole pigment, has long been recognised for its antimicrobial property. However, the physiological contribution of prodigiosin to the survival of its producing hosts still remains undefined. Hence, the aim of this study was to investigate the biological role of prodigiosin from *Serratia marcescens*, particularly in microbial competition through its antimicrobial activity, towards the growth and secreted virulence factors of four clinical pathogenic bacteria (methicillin-resistant *Staphylococcus aureus* (MRSA), *Enterococcus faecalis*, *Salmonella enterica* serovar Typhimurium and *Pseudomonas aeruginosa*) as well as *Staphylococcus aureus* and *Escherichia coli*. Prodigiosin was first extracted from *S*. *marcescens* and its purity confirmed by absorption spectrum, high performance liquid chromatography (HPLC) and liquid chromatography-tandem mass spectrophotometry (LC-MS/MS). The extracted prodigiosin was antagonistic towards all the tested bacteria. A disc-diffusion assay showed that prodigiosin is more selective towards Gram-positive bacteria and inhibited the growth of MRSA, *S*. *aureus* and *E*. *faecalis* and Gram-negative *E*. *coli*. A minimum inhibitory concentration of 10 μg/μL of prodigiosin was required to inhibit the growth of *S*. *aureus*, *E*. *coli* and *E*. *faecalis* whereas > 10 μg/μL was required to inhibit MRSA growth. We further assessed the effect of prodigiosin towards bacterial virulence factors such as haemolysin and production of protease as well as on biofilm formation. Prodigiosin did not inhibit haemolysis activity of clinically associated bacteria but was able to reduce protease activity for MRSA, *E*. *coli* and *E*. *faecalis* as well as decrease *E*. *faecalis*, *Salmonella* Typhimurium and *E*. *coli* biofilm formation. Results of this study show that in addition to its role in inhibiting bacterial growth, prodigiosin also inhibits the bacterial virulence factor protease production and biofilm formation, two strategies employed by bacteria in response to microbial competition. As clinical pathogens were more resistant to prodigiosin, we propose that prodigiosin is physiologically important for *S*. *marcescens* to compete against other bacteria in its natural soil and surface water environments.

## Introduction

**Fig 1:**
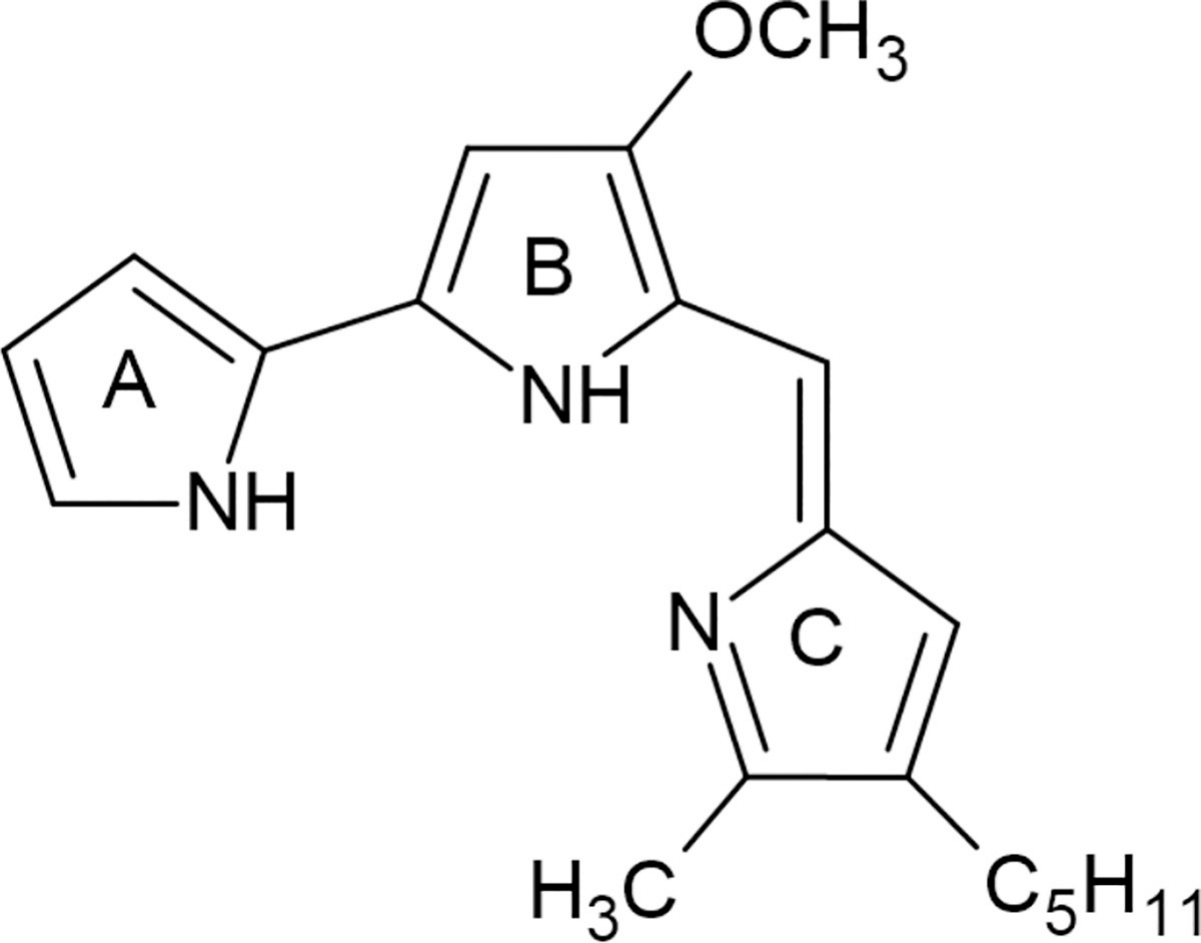
The structure of prodigiosin. The three rings, A, B and C made up the tripyrrole structure of prodigiosin (Adapted from Williamson et al. [3]).

Prodigiosin is a red tripyrrole bacterial pigment normally secreted by the human pathogen, *Serratia marcescens*, as a secondary metabolite during the bacterial idiophase [1]. In *S*. *marcescens*, prodigiosin is synthesised by the prodigiosin biosynthesising gene cluster (*pig* cluster) of ~20 kb and a bifurcated pathway acts in concert to produce two key intermediates, 2-methyl-3-n-amylpyrrole (MAP) and 4-methoxy-2,2’-bipyrrole-5-carbaldehyde (MBC), which are then condensed by PigC to produce prodigiosin [2, 3]. Prodigiosin consists of three pyrrole rings (A, B and C). Both the A and B rings are bridged in a bipyrrole unit whilst the B and C rings are attached in a dipyrrin [4]. The monopyrrole moiety (C ring) is connected to the methoxy bipyrrole moiety (A and B rings) by a methylene bridge [5]. The tripyrrole structure of prodigiosin is illustrated in Fig 1.

Prodigiosin has long been a subject of research interest owing to its anticancer [6], antimalarial [7], antifungal [8] and antibacterial [9, 10] properties. As a typical secondary metabolite, prodigiosin has no clearly defined physiological function in pigmented *S*. *marcescens* [11]. Some of the possible functions of prodigiosin include host protection from challenges within the natural environment [12, 13] and/or to facilitate ecological bacterial dispersal [14]. Another possible physiological role that prodigiosin plays is in interspecies competition whereby prodigiosin inhibits growth of a wide spectrum of Gram-positive and Gram-negative bacteria that grow in similar environments [15, 16]. While little is known about the biological role of prodigiosin production, Haddix et al. [17] suggested that prodigiosin acts as a mediator of energy spilling reactions in *S*. *marcescens* whilst other studies have hinted in light energy storage [18] or ultraviolet (UV) protection [19]. Synthesis of the antimicrobial red pigment is dependent on environmental conditions and may be regulated by more favourable conditions to achieve microbial ecology dominance over competitor species [20]. Given the huge amount of energy invested in prodigiosin synthesis [2], this metabolite is undoubtedly important in the survival of *S*. *marcescens*, particularly during interspecies competition.

Previously, the antimicrobial potency of prodigiosin was attributed to three mechanisms: bacterial deoxyribonucleic acid (DNA) cleavage, cell cycle inhibition and pH modulation [21]. Recently, Suryawanshi et al. [22] proposed that prodigiosin is a hydrophobic stressor able to disrupt the plasma membrane of competitor bacteria via a chaotropic-mediated mode-of-action. Other mechanisms proposed include phototoxicity [23] and formation of reactive oxygen species (ROS) [24]. Furthermore, prodigiosin was reported to induce autolysins production in actively growing *Bacillus subtilis* and other *Bacillus* species [25]. Nevertheless, it is possible that the reported prodigiosin antimicrobial property is not solely attributed to the disruption of single cell targets, but in turn, may have a pleiotropic effect on bacterial physiology such as the integrity of the outer membrane, cell respiration as well as the synthesis of bacterial RNA and proteins [26] of the competitor bacteria. In this study, our aim was to further understand antibacterial mechanisms of prodigiosin and how this metabolite aids in *S*. *marcescens* survival in the presence of potential competitor bacteria.

To elucidate the possible biological role of prodigiosin in microbial interactions, especially during interspecies competition, the antibacterial effect of prodigiosin towards three Gram-positive (methicillin-resistant *Staphylococcus aureus* (MRSA), *Staphylococcus aureus*, *Enterococcus faecalis*) and three Gram-negative (*Escherichia coli*, *Salmonella enterica* serovar Typhimurium, *Pseudomonas aeruginosa*) bacteria were tested. Out of the six selected bacteria, MRSA, *E*. *faecalis*, *Salmonella* Typhimurium and *P*. *aeruginosa* are clinical strains whereas the *S*. *aureus* and *E*. *coli* used are standard laboratory strains. Prodigiosin was first produced and extracted using solvent method from *S*. *marcescens* and identified using absorption spectrum, high performance liquid chromatography (HPLC) and liquid chromatography-tandem mass spectrophotometry (LC-MS/MS). The antibacterial property of the crude extracted prodigiosin towards the six bacteria was then evaluated. Growth inhibition of prodigiosin-treated bacterial cultures was examined using disc-diffusion and minimum inhibitory concentration (MIC) assays. The pathogens were then treated with prodigiosin to determine its effect on bacterial haemolysin and protease activity and formation of biofilm. The results suggest that prodigiosin is advantageous for the survival of *S*. *marcescens* during microbial competition in a given environment such as in soil and surface water.

## Materials and methods

### Bacterial strains and culture media

*Serratia marcescens* Bizio ATCC^®^ 274^TM^ (Sma 274) and methicillin-resistant *Staphylococcus aureus* (MRSA) ATCC^®^ 33591^TM^ were obtained from the American Type Culture Collection (ATCC). *Staphylococcus aureus* NCTC 8325–4, *Escherichia coli* OP50, *Pseudomonas aeruginosa* PA14, *Salmonella enterica* serovar Typhimurium SL1344 and *Enterococcus faecalis* V583 were obtained from the Pathogen Laboratory, Universiti Kebangsaan Malaysia (UKM). Sma 274 was routinely cultivated on peptone glycerol (PG) media (pH 7) at 28°C for 16 hours whereas the remaining bacteria strains were cultivated at 37°C for 16 hours. MRSA and *S*. *aureus* were propagated on trypticase soy (TS) media (Oxoid, England) with 30 μg/mL nalidixic acid (Sigma-Aldrich, USA) whilst *E*. *coli* and *Salmonella* Typhimurium were cultured on Luria-Bertani (LB) media (Lennox, USA), supplemented with streptomycin (100 μg/mL) (Calbiochem, USA). *P*. *aeruginosa* was cultured in King’s B media supplemented with 100 μg/mL rifampicin (Calbiochem, USA) whilst *E*. *faecalis* was cultivated in Brain-Heart-Infusion (BHI) media (Lennox, USA) supplemented with 50 μg/mL kanamycin (Calbiochem, USA).

PG broth (PGB) was made up of (/L) 5 g peptone and 10 mL glycerol. King’s B broth contained (/L) 20 g peptone, 1.5 mM K_2_HPO_4_ and 6 mM MgSO_4_. To prepare PG agar (PGA) and King’s B agar, 15 g and 20 g bacteriological agar (Pronadisa, Spain) was added into 1 litre of PGB and King’s B broth, respectively. For the haemolysis assay, the 5% blood TS agar (TSA) contained (/L) 500 mL of fresh sheep blood, 6 mL of ethylenediaminetetraacetic acid (EDTA) (Ohio, USA) and 1× phosphate buffered saline (PBS) solution (pH 7.0 ± 0.2) (Sigma-Aldrich, USA). For the proteolysis assay, the 3% skim milk agar contained (/L) 30 g skim milk powder (Sigma-Aldrich, USA) and 35 g LB agar (Lennox, USA).

### Prodigiosin production by *Serratia marcescens*

Sma 274 was cultured on PG agar and incubated at 28°C for 16 hours. A single bacterial colony was picked and inoculated into 10 mL PGB, followed by incubation at 28°C for 16 hours at 250 rpm in the Innova^TM^ 4200 Incubator Shaker (New Brunswick Scientific, USA). The optical reading of the culture was then normalised to 0.4 and the normalised bacterial culture was diluted 100× with PG broth and incubated at 28°C for 96 hours. After the 96-hour incubation period, 1 mL of the *S*. *marcescens* culture was used to estimate prodigiosin production using the formula below [27]:

$$\begin{array}{l} Prodigiosin\;units/cell=\frac{(\left[{\mathrm{OD}}_{499}–(1.381\times {\mathrm{OD}}_{620})\right])\times 1000}{{\mathrm{OD}}_{620}}\\ \;\mathrm{Where},\;{\mathrm{OD}}_{499}=\mathrm{pigment}\;\mathrm{absorption}\;\mathrm{in}\;\mathrm{culture}\\ \;{\mathrm{OD}}_{620}=\mathrm{bacterial}\;\mathrm{culture}\;\mathrm{absorption}\\ \;1.381=\mathrm{constant} \end{array}$$

Prodigiosin in bacterial culture is detected by observing a single peak at 499 nm (OD_499_) [27]. The value (1.381 × OD_620_) represents the number of bacterial cells so that OD_499_-(1.381× OD_620_) reflects the absorption of prodigiosin. The absorbance value of prodigiosin is then divided by the bacterial cell absorbance value (OD_620_) to express prodigiosin units on a per cell basis and a factor of 1000 is included in the formula to avoid working with small numbers (<1).

### Extraction of prodigiosin using acidic solvent

Prodigiosin was extracted using 95% methanol as previously described [28] with modifications. Briefly, 100 mL of the 96-hour incubated *S*. *marcescens* culture was centrifuged at 10,000 ×*g* for 10 minutes. The supernatant was discarded and pellet resuspended with 100 mL of 95% methanol. Thereafter, the bacterial suspension was vortexed, followed by centrifugation at 10,000 ×*g* for 30 minutes to remove undissolved components. The supernatant was measured at wavelengths between 200–700 nm using the Ultrospec 10 spectrophotometer. The absorption profile was recorded to identify the presence of prodigiosin. The remaining supernatant was vacuum-dried and the dry mass of prodigiosin was determined before it was dissolved in 95% methanol.

### Identification of prodigiosin in the extracted sample

Reversed-phase high performance liquid chromatography (HPLC) was performed to confirm the presence and purity of prodigiosin in the crude extract. HPLC was performed on a Waters Alliance 2695/2795 Separation Unit with 2996 Photodiode Array Detector (PDA) with a Kinetex^®^ C18 LC column (4.6 × 250 mm and 5 μm) (Phenomenex, USA) for 15 minutes with an injection volume of 20 μL of crude prodigiosin extract (250 μg/μL). The mobile phase was methanol:water:acetonitrile (73:20:7) [29] in 0.2% acetic acid [30] and the detection wavelength was 535 nm [14]. Due to the prohibitive costs of a pure prodigiosin standard, the HPLC chromatogram was compared to that reported by Lin et al. [29].

To further validate the presence and purity of prodigiosin, the extract was subjected to untargeted reversed-phase HPLC coupled with tandem mass spectrophotometry (LC-MS/MS) using the Agilent 6550 iFunnel Quadrupole Time-of-Flight (Q-TOF) LC/MS system (Agilent Technologies, USA). Briefly, ten microliters of the crude prodigiosin extract (10 μg/mL) was separated using a Zorbax^®^ Eclipse Plus C18 Rapid Resolution column (4.6 × 100 mm and 3.5 μm) (Agilent Technologies, USA). The column temperature was maintained at 40°C and separation of the extract was facilitated with milliQ water containing 0.1% (*v/v*) formic acid as solvent A and acetonitrile containing 0.1% (*v/v*) formic acid as solvent B. The flow rate was 0.3 mL per minute (mL/min) with gradient developed as follows: 60% of solvent A from 0 to 0.5 min for loading, linear gradient from 20 to 80% (solvent B) from 0.5 to 14 min and 0–100% (solvent B) from 14 to 15 min [31]. The elutes were then subjected to a capillary voltage of 3.5 kV, source temperature of 200°C, nebuliser pressure of 35 psi, desolvation nitrogen gas at flow rate of 11 L/min, desolvation temperature at 350°C and collision energy at 0 eV. Data acquisition in the positive mode was performed by MS scanning through the mass-to-charge (*m/z*) range of 40–1700. The LC-MS/MS data was then processed using MassHunter Personal Compound Database and Library Manager Software (Agilent Technologies, USA).

### Antimicrobial assay of prodigiosin

The antimicrobial property of prodigiosin was evaluated using the Kirby-Bauer standardised disc-diffusion assay [32] on Mueller-Hinton agar (MHA) (Le Chair, France). Previously, our preliminary results showed that crude prodigiosin extract exhibited antimicrobial activity at 500 μg/μL (data not shown). Hence, in this study, we tested the antimicrobial activity of prodigiosin at 500 μg/μL and 250 μg/μL. Sterile Whatmann filter paper was used as discs (6 mm) and impregnated with 35 μg/μL chloramphenicol (positive control) (Sigma-Aldrich, USA), 99% ethanol (negative control; used as solvent for chloramphenicol), 95% methanol (negative control, used as solvent for solubilising prodigiosin extract) and either 250 μg/μL or 500 μg/μL prodigiosin extract. Each bacterial species (MRSA, *S*. *aureus*, *E*. *faecalis*, *E*. *coli*, *Salmonella* Typhimurium and *P*. *aeruginosa*) was individually inoculated into 5 mL of their respective broth and cultured at 37°C for 16 hours at 250 rpm using the Innova^TM^ Incubator Shaker (New Brunswick Scientific, USA). The bacterial cultures were normalised to OD_600_ = 0.4 using their respective broths corresponding to approximately 10^8^ CFU/mL. A total of 100 μL of each normalised culture was then spread onto MHA using sterile glass beads and allowed to dry before the five impregnated discs were aseptically placed onto the dried bacterial lawn using sterile forceps. The plates were then incubated at 37°C for 24 hours. Any zones of inhibition formed around the discs were then measured and recorded. For each bacterial sample, three biological replicates were performed.

### Minimum inhibitory concentration (MIC) of prodigiosin

Prodigiosin from the stock was diluted to 20 μg/μL and 100 μL of the diluted prodigiosin was added into wells of a 96-well microtiter plate (Greiner Bio-One, Austria). An overnight culture of each bacterial species (MRSA, *S*. *aureus*, *E*. *faecalis*, *E*. *coli*, *Salmonella* Typhimurium and *P*. *aeruginosa*) was normalised to OD_600_ of 0.4 (~10^8^ CFU/mL) using their respective broth. The normalised bacterial culture (100 μL) was then added into triplicate wells pre-added with prodigiosin (20 μg/μL) resulting in a final prodigiosin working concentration of 10 μg/μL. Across the rows of wells, prodigiosin was serially diluted by two-fold to 5, 2.5, 1.25, 0.63, 0.313, 0.156 and 0.078 μg/μL [33]. In this assay, bacterial cultures treated with chloramphenicol (70 μg/mL) were used as the positive control whereas untreated bacterial cultures were used as the negative control. The plates were incubated at 37°C for 48 hours and the wells containing the prodigiosin treated bacterial cultures were observed to determine the minimum concentration of prodigiosin required to inhibit growth of the respective tested bacteria.

### Haemolysis assay

The haemolysis assay measures the release of haemoglobin when erythrocytes are treated with bacterial haemolysin. In this study, the assay was performed on TSA with 5% fresh sheep blood to determine if prodigiosin inhibits haemolysin secreted by the bacterial pathogens being tested. Approximately 10^5^ CFU (10 μL) of each bacterial sample pre-treated with 500 μg/μL prodigiosin was spotted onto the blood agar. For each tested pathogen, the positive control used was untreated bacterial culture whilst the negative control used was their respective broth. Bacterial cultures treated with 95% methanol were also included in this assay. The plates were incubated at 37°C for 48 hours. Triplicates were performed for each tested pathogen and the formation of clear zones on the blood agar was recorded.

### Proteolysis assay

This assay was performed to investigate the effectiveness of prodigiosin in inhibiting proteases secreted by the tested pathogens using 3% skim milk agar. The agar contains casein as the main ingredient and proteases secreted by the bacteria will hydrolyse the casein to form visible inhibition zones. Approximately 10^5^ CFU (10 μL) of each bacterial sample pre-treated with 500 μg/μL prodigiosin was spotted onto the skim milk agar. The positive control used in this assay was untreated bacteria whereas 95% methanol treated bacteria was used as solvent control. The plates were then incubated at 37°C for 24 hours [34]. Triplicate assays were performed for each tested pathogen. The surface area of the inhibition halo zones was measured using the ellipse formula shown below:

A=πab

Where, A = Surface area, π = 3.142, a = y-axis radius, b = x-axis radius

### Biofilm assay

To evaluate if prodigiosin can inhibit bacterial biofilm formation, the biofilm assay was performed [35]. Briefly, overnight bacterial cultures were normalised to OD_600_ = 0.4 (10^8^ CFU/mL) using their respective broth. The diluted bacterial cultures were then treated with 500 μg/μL of prodigiosin and added into 96-well microtiter plates (Greiner Bio-One, Austria) and incubated at 37°C for 48 hours. For each bacterial sample, the negative control used was their respective broth while the positive control used was *S*. *aureus*, a known high biofilm former [36]. After the 48-hour incubation period, each well was washed three times with 200 μL of 1× PBS after which 200 μL of 99% methanol was added into each well and left for 15 minutes at room temperature. Methanol was then discarded and the plate was allowed to dry for 15 minutes. Then, 200 μL of 0.5% crystal violet (Sigma-Aldrich, USA) was added into each well and left at room temperature for 5 minutes to stain the bacterial biofilm. Excess crystal violet was then washed away using distilled water and plates were left to dry completely at room temperature. Finally, 95% ethanol was used to dissolve the crystal violet bound to the biofilm. Optical readings of the dissolved crystal violet at 570 nm were measured on the Magellan^TM^ software using the Sunrise absorbance microplate reader (Tecan, Switzerland).

## Results

### Quantification and identification of extracted prodigiosin

**Fig 2:**
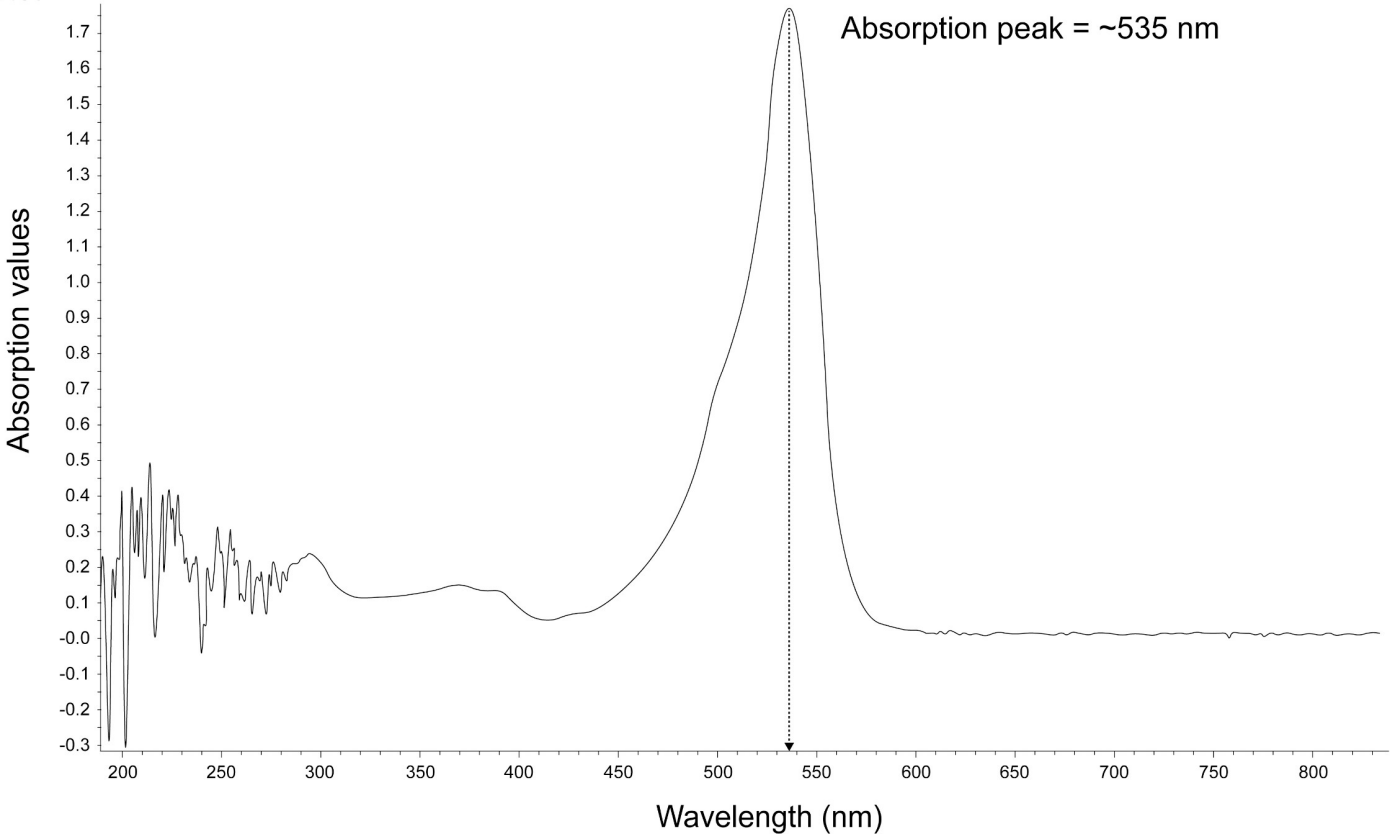
Absorbance profile (200–700 nm) of the crude extracted prodigiosin. Red prodigiosin in the extracted sample is detectable at a maximum of 535 nm.

To investigate the physiological role of prodigiosin, we used natural crude prodigiosin extract instead of a synthetic prodigiosin. Previously we had determined that prodigiosin production by *S*. *marcescens* Sma 274 was optimal when the bacterium was cultured in PGB at 28°C, pH 7 and incubated for 96 hours [37]. In this present study, under these culture conditions, the amount of prodigiosin produced was approximately 891.61 units/cell. In an acidic solvent, prodigiosin appears as red color usually detectable at 535 nm [38]. Fig 2 shows the absorbance profile of the crude prodigiosin extracted from *S*. *marcescens* Sma 274 cultured in PGB where a sharp spectral peak at 535 nm signified the presence of prodigiosin, confirming the presence of prodigiosin in the extracted sample. The single peak at 535 nm and the absence of other peaks or disturbances also attested to the purity of prodigiosin in the extracted crude sample. No compounds were detected in the negative control, 95% methanol. Thereafter, the dry mass of prodigiosin was dissolved in 95% methanol to a final stock concentration of 800 μg/μL.

**Fig 3:**
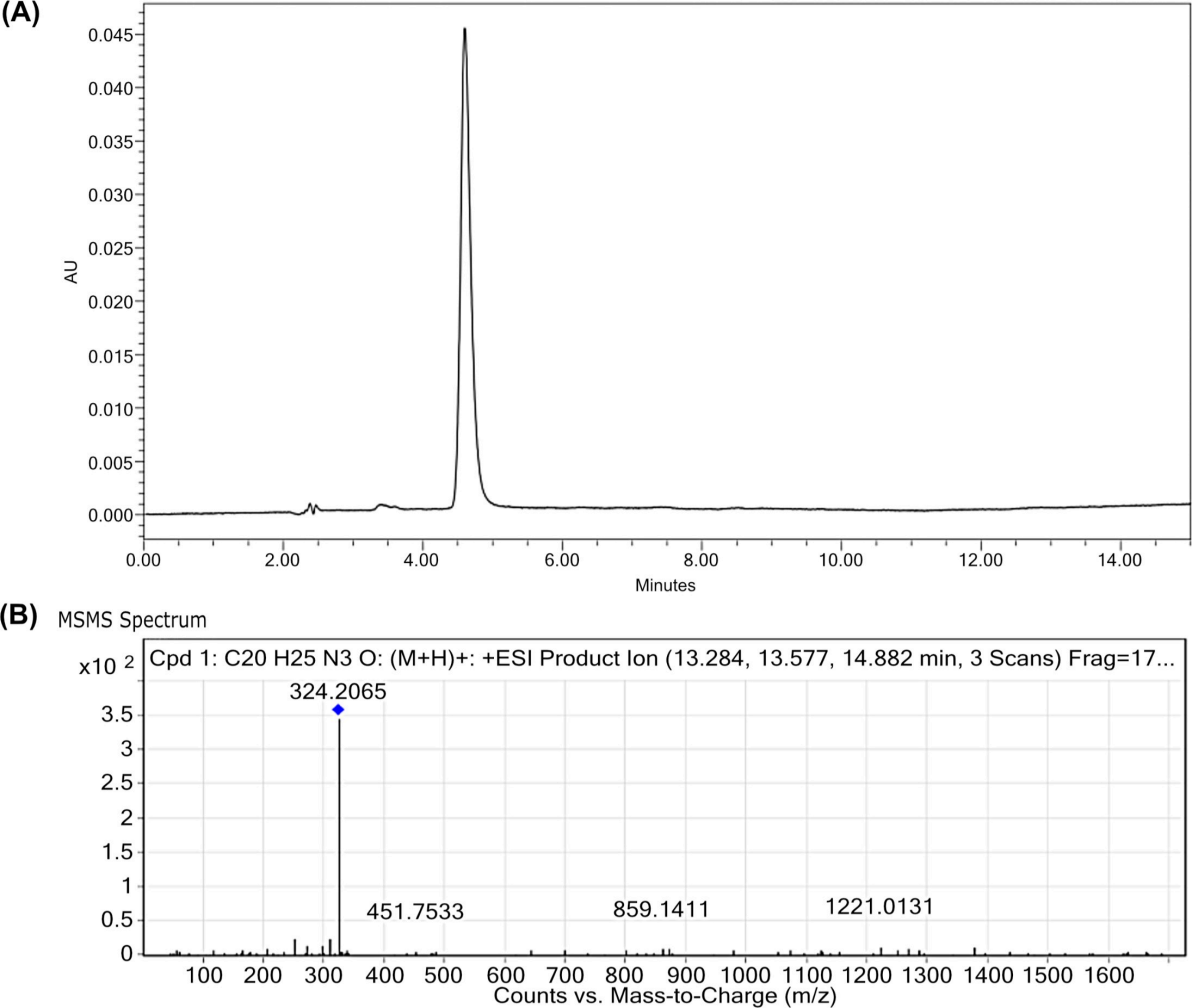
Identification of prodigiosin in the crude extract. Prodigiosin is detected at the (A) retention time of ~4.75 minutes by HPLC. (B) The molecular weight of prodigiosin (324.2068 g mol^-1^) was detected from the LC-MS/MS analysis.

To further identify and confirm the presence of prodigiosin in the extracted sample, HPLC and LC-MS/MS were carried out. Prodigiosin was detected by the HPLC where only a single peak was observed at 535 nm with a retention time of ~4.75 minutes (Fig 3A). In the absence of a pure prodigiosin standard, the retention time of 4.75 minutes was compared to the published retention time of 6 minutes for prodigiosin standards [29]. The slight difference in the retention times in this study and Lin et al. [29] could be attributed to the different make of HPLC machines and columns used. Hence, to validate the purity of prodigiosin in our sample, the extract was subjected to LC-MS/MS. Results from LC-MS/MS showed that the highest peak was detected at *m/z* = 324.2065 g/mol (Fig 3B), corresponding to the molecular weight of prodigiosin ion ([C_20_H_25_N_3_O+H])^+^ with a retention time of 13.5 minutes. Taken together, these results confirm that prodigiosin is present as a predominant compound in the crude sample. Hence, the prodigiosin within the crude extract is of a relatively high purity and no further purification steps were carried out for the sample.

### Prodigiosin is antagonistic towards the growth of selected pathogenic bacteria

**Fig 4:**
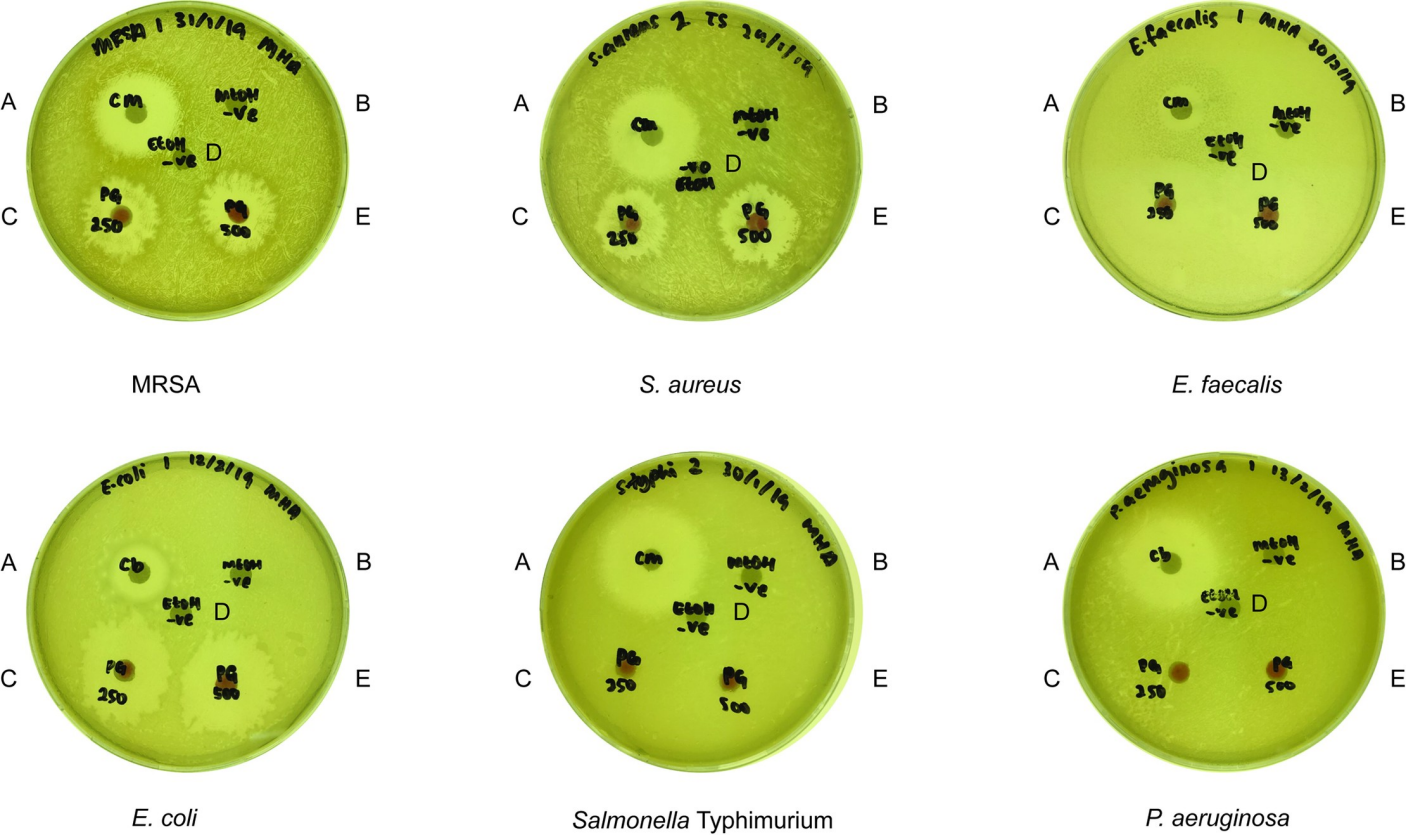
Disc-diffusion assay of selected pathogenic bacteria after 24 hours exposure to prodigiosin. The antimicrobial property of prodigiosin was evaluated using the Kirby-Bauer standardised disc-diffusion assay. Filter paper discs were impregnated with (A) 35 μg/μL chloramphenicol (positive control), (B) 95% methanol (negative control, used as solvent for solubilising prodigiosin extract), (C) 250 μg/μL crude prodigiosin (D) 500 μg/μL crude prodigiosin and (E) 99% ethanol (negative control; used as solvent for chloramphenicol).

In the disc-diffusion assay, three Gram-positive (MRSA, *S*. *aureus* and *E*. *faecalis*) and three Gram-negative (*E*. *coli*, *Salmonella* Typhimurium and *P*. *aeruginosa*) bacteria were selected to test the effectiveness of prodigiosin in inhibiting bacterial growth. Chloramphenicol (35 μg/μL; positive control) successfully inhibited the growth of all the tested bacteria while no inhibition zones were observed with the negative controls. Of the six selected bacteria treated with prodigiosin, three Gram-positive (MRSA, *S*. *aureus*, *E*. *faecalis*) and one Gram-negative (*E*. *coli*) bacteria were inhibited by prodigiosin at 250 μg/μL and 500 μg/μL. The growth of both *P*. *aeruginosa* and *Salmonella* Typhimurium was not negatively affected by prodigiosin at these concentrations (Fig 4; Table 1; S1 Table in S1 Appendix). Therefore, the MIC values of crude prodigiosin extract on both *P*. *aeruginosa* and *Salmonella* Typhimurium were not evaluated. The MIC of crude extracted prodigiosin on *S*. *aureus*, *E*. *faecalis* and *E*. *coli* was ≥ 10 μg/μL whereas > 10 μg/μL was required to inhibit the visible growth of MRSA (Table 2). A higher MIC value of prodigiosin needed to inhibit MRSA was expected since MRSA is a clinical strain, proposing that prodigiosin has not developed high affinity towards most of the clinical pathogens (MRSA, *Salmonella* Typhimurium and *P*. *aeruginosa*) tested and suggests that prodigiosin has a role in thwarting *S*. *marcescens*’ competitors that are generally found in the same environment.

**Table 1:** Average diameter of inhibition zones of the bacteria tested after 24 hours. Zones of inhibition formed around the discs impregnated with either 250 μg/μL or 500 μg/μL crude prodigiosin extract were measured and recorded. Inhibition of bacterial growth was interpreted as a positive inhibition when compared to the corresponding inhibition zone from chloramphenicol treatment. Data are presented as averages and standard deviations (n = 3).

Bacteria	Diameter of inhibition zone (mm)				
	Positive control	Negative control		+ Prodigiosin	
	Chloramphenicol (35 μg/μL)	EtOH	MeOH	250 μg/μL	500 μg/μL
Methicillin-resistant *Staphylococcus aureus*	23 ± 0.33	0	0	20 ± 0.33	21 ± 0.00
*Staphylococcus aureus*	27 ± 1.67	0	0	20 ± 0.00	22 ± 0.33
*Enterococcus faecalis*	19 ± 0.67	0	0	20 ± 0.88	20 ± 0.33
*Escherichia coli*	29 ± 0.00	0	0	22 ± 0.41	27 ± 0.82
*Salmonella* Typhimurium	28 ± 0.67	0	0	0	0
*Pseudomonas aeruginosa*	26 ± 0.41	0	0	0	0

EtOH, ethanol; MeOH, methanol

**Table 2:** The minimum inhibitory concentration (MIC) values of prodigiosin on the tested bacteria after 48 hours prodigiosin treatment. The minimum concentration of prodigiosin required to inhibit visible microbial growth was determined by observing the turbidity of the culture in the wells. Non-turbid (clear) cultures were interpreted as positive bacterial growth inhibition when compared to the corresponding inhibition following chloramphenicol treatment (n = 3).

Bacteria	Minimum inhibitory concentration (μg/μL)
Methicillin-resistant *Staphylococcus aureus*	> 10
*Staphylococcus aureus*	10
*Enterococcus faecalis*	10
*Escherichia coli*	10

### Prodigiosin does not inhibit haemolysin activity

Haemolysin is secreted by a number of bacterial isolates to acquire nutrients from lysed erythrocytes of the infected host. Haemolysis is categorised into complete (β-haemolysis), incomplete (α-haemolysis) and no destruction (γ-haemolysis) of erythrocytes [39]. Bacterial colonies that form yellow-coloured lytic zones on blood agar have β-haemolytic activity due to the complete lysis of erythrocytes [40] whereas greenish discolouration surrounding the bacterial colonies indicates α-haemolytic activity [41]. Bacteria that do not induce haemolysis are categorised in the γ-haemolysis group [39].

**Fig 5:**
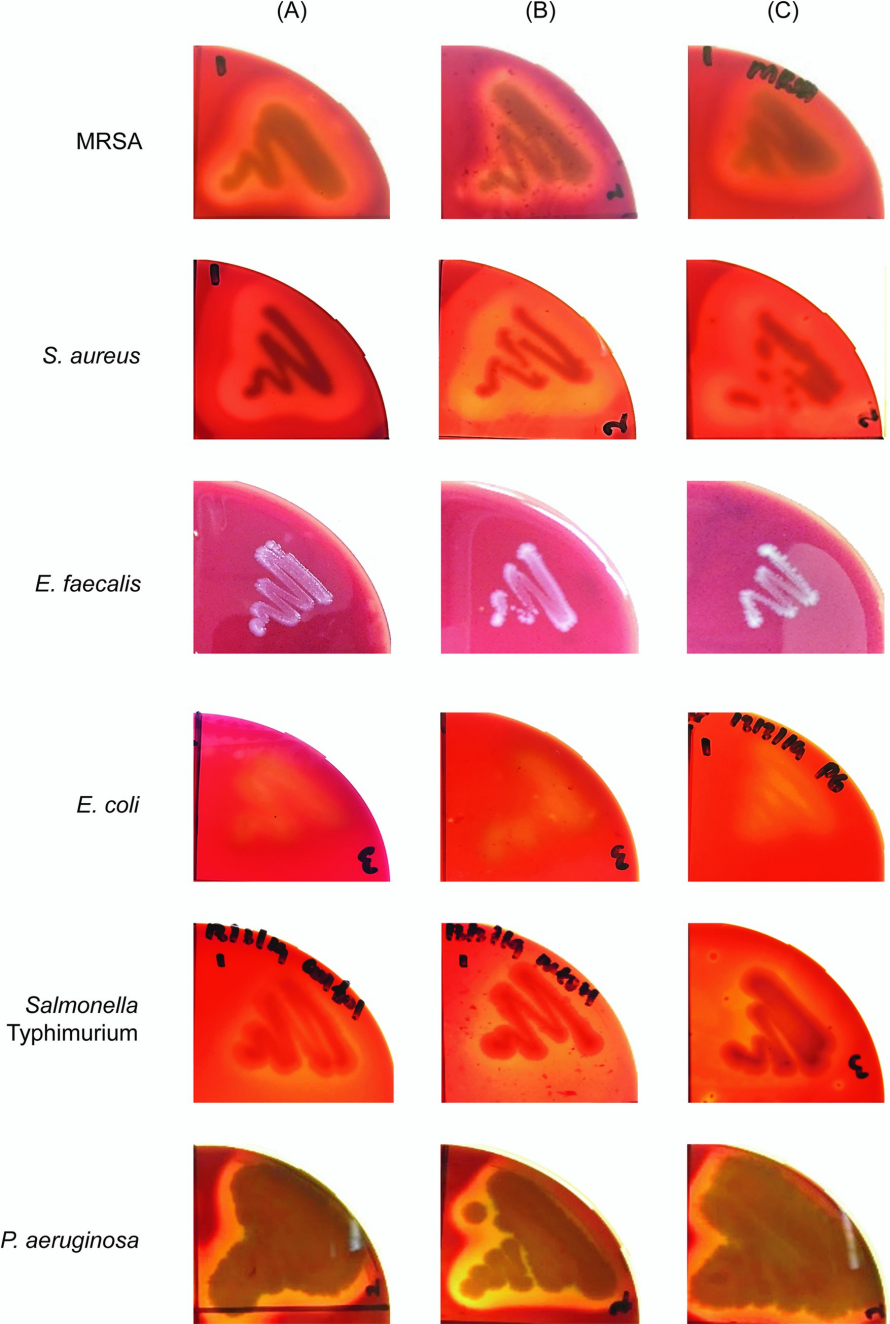
Haemolytic activity of the selected bacteria in the presence and absence of prodigiosin. Individual bacteria (10^5^ CFU), either untreated or pre-treated with 500 μg/μL prodigiosin were spotted onto blood agar. For each tested pathogen, the positive control used was (A) untreated bacterial culture whilst the solvent control included in this assay was (B) bacterial cultures treated with 95% methanol. Plates were incubated at 37°C for 48 hours. Triplicates were performed for each tested pathogen and the observation of haemolysis by (C) prodigiosin-treated bacteria was recorded.

We noted that clear zones were observed for both untreated bacterial cultures and solvent control over the period of the assay (Fig 5) indicating that 95% methanol does not interfere with the bacterial haemolytic activities. Both prodigiosin-treated and untreated *E*. *faecalis* cultures did not cause haemolysis, indicating that *E*. *faecalis* V583 belongs to the γ-haemolysis group even though other strains of *E*. *faecalis* are known to exhibit β-haemolytic activity [25]. *E*. *coli* presented α-haemolysis [42] in both prodigiosin-treated and untreated cultures while *Salmonella* Typhimurium demonstrated α-haemolysis which is similar to the *Salmonella* Pathogenicity Island (SPI)-1 type III secretion-dependent haemolytic activity exhibited by *Salmonella* Typhimurium SL1344 [43]. Previously, it was reported that MRSA [44], *S*. *aureus* [45] and *P*. *aeruginosa* [46] exhibit β-haemolysis. Indeed, complete lysis of erythrocytes was observed in both prodigiosin-treated and untreated cultures of these bacteria with *S*. *aureus* exhibiting double haemolysis due to the presence of both α- and β-haemolysins [47] (Fig 5). Nonetheless, for the bacterial cultures treated with prodigiosin, there was no noticeable reduction in haemolytic activity when compared to the untreated bacteria (Fig 5A and 5C). This is not altogether surprising as haemolysin is most likely only secreted by pathogenic bacteria during an active infection of a host and would not pose a threat to *S*. *marcescens* in the environment.

### Prodigiosin inhibits protease activity

**Fig 6:**
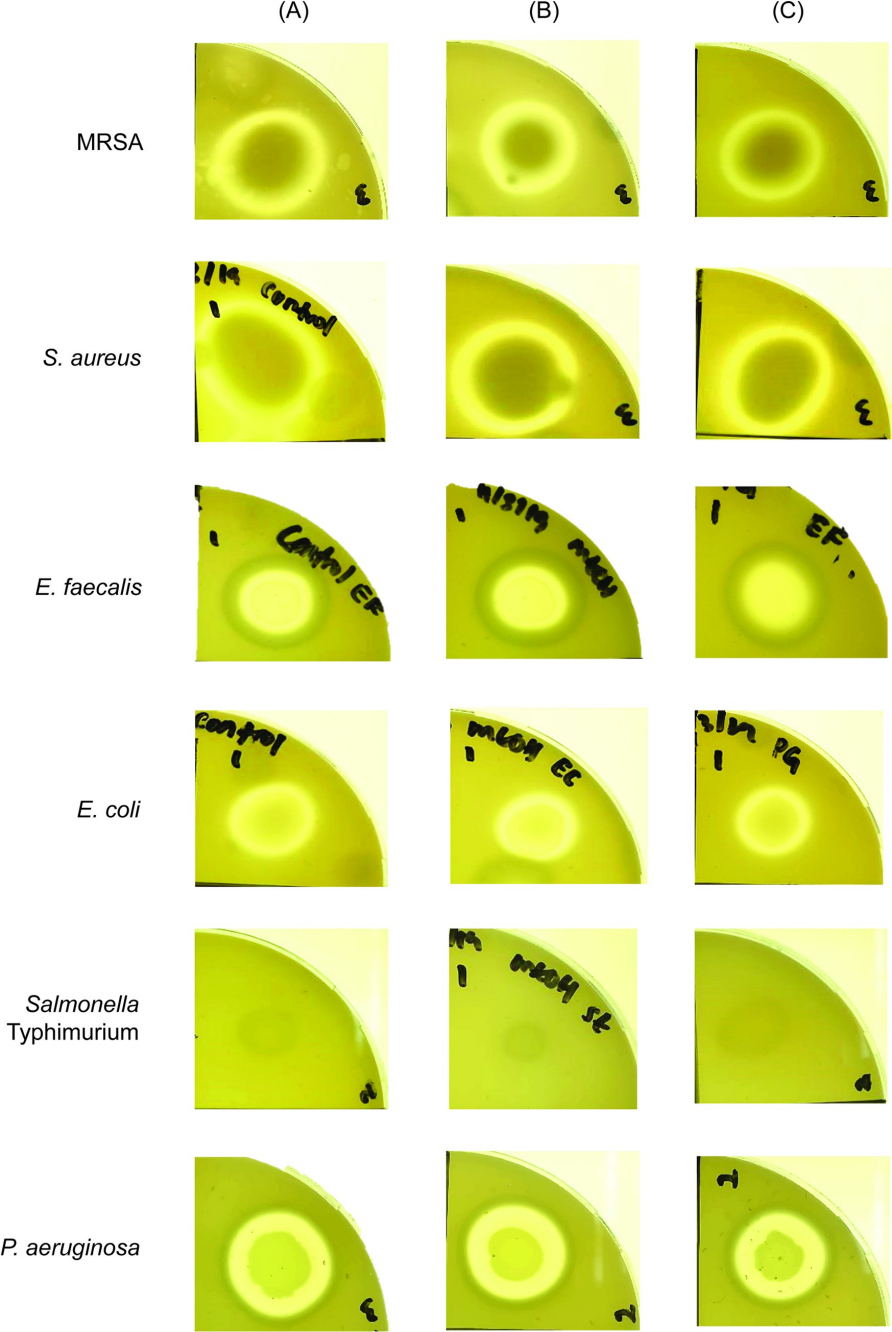
Proteolytic zones of the selected pathogenic bacteria. Individual untreated and prodigiosin pre-treated bacteria were spotted onto skim milk agar. The positive control used was (A) untreated bacterial culture whilst the solvent control included in this assay was (B) bacterial cultures treated with 95% methanol. Plates were incubated at 37°C for 24 hours. Triplicates were performed for each tested pathogen and the observation of proteolysis by (C) prodigiosin-treated bacteria was recorded.

Proteases are important virulence factors secreted by pathogenic bacteria that degrade proteins with vital roles in host innate immunity or immunoglobulins, thereby facilitating the successful colonisation and dissemination of the infecting bacteria [48]. We assessed the secretion of proteases by the selected bacteria treated with prodigiosin. Proteolytic zones observed for MRSA, *S*. *aureus*, *E*. *faecalis*, *E*. *coli* and *P*. *aeruginosa* treated with prodigiosin were smaller compared to the untreated bacterial cultures, indicating that prodigiosin interfered with proteolysis or protease secretion by these bacteria. The anti-proteolytic effect of prodigiosin was most significant for both MRSA and *E*. *coli* (p<0.05) followed by *E*. *faecalis*, *P*. *aeruginosa* and *S*. *aureus*. No inhibition zones were observed for treated and untreated *Salmonella* Typhimurium suggesting that this strain is incapable of secreting proteases (Fig 6; Table 3; S2 Table in S1 Appendix).

**Table 3:** Average area of proteolytic zones following prodigiosin treatment on the bacterial cultures for 24 hours. Data are presented as averages and standard deviations (n = 3).

Bacteria	Area of inhibition (mm^2^)			*P*-value*
	MeOH control	- Prodigiosin	+ Prodigiosin	
Methicillin-resistant *Staphylococcus aureus*	24 ± 0.88	28 ± 1.55	22 ± 0.33	0.01
*Staphylococcus aureus*	26 ± 0.33	29 ± 2.96	24 ± 0.50	0.33
*Enterococcus faecalis*	24 ± 0.33	25 ± 0.33	23 ± 0.33	0.05
*Escherichia coli*	11 ± 0.33	12 ± 0.33	10 ± 0.33	0.01
*Salmonella* Typhimurium	0 ± 0.00	0 ± 0.00	0 ± 0.00	-
*Pseudomonas aeruginosa*	21 ± 0.88	22 ± 0.33	20 ± 0.33	0.10

MeOH, methanol

*p values were calculated using the Student’s t-test

### Prodigiosin as an anti-biofilm agent

Pathogenic bacteria form biofilms that protect the bacteria from antibiotics and other antimicrobials [49] as well as in response to harsh environments [50]. As prodigiosin may contribute to bacteria dispersal [25], the effect of prodigiosin (500 μg/μL) on biofilm formation was assessed for the six selected bacteria. The positive control used was *S*. *aureus* because it is a known high biofilm former [36] whereas the negative control used was broth (TS broth for both MRSA and *S*. *aureus*; LB broth for both *E*. *coli* and *Salmonella* Typhimurium; King’s B broth for *P*. *aeruginosa*; BHI broth for *E*. *facealis*). We noted a significant reduction in biofilm formation for prodigiosin-treated *E*. *faecalis* and *Salmonella* Typhimurium (p<0.05) when compared to the respective untreated bacteria. Surprisingly, *S*. *aureus* (p<0.05) and *P*. *aeruginosa* responded to the presence of prodigiosin by increasing biofilm formation. The amount of biofilm formed by MRSA did not change in the presence of prodigiosin (Table 4; S3 Table in S1 Appendix).

**Table 4:** Biofilm formation by prodigiosin-treated and untreated bacterial cultures after 48 hours incubation. Data are presented as averages and standard deviations (n = 3).

Bacteria	Absorbance readings at 570 nm		*P*-value*
	- Prodigiosin	+ Prodigiosin	
Methicillin-resistant *Staphylococcus aureus*	0.20 ± 0.04	0.20 ± 0.01	0.94
*Staphylococcus aureus*	0.28 ± 0.07	0.73 ± 0.09	0.01
*Enterococcus faecalis*	0.75 ± 0.07	0.47 ± 0.04	0.01
*Escherichia coli*	0.14 ± 0.01	0.11 ± 0.01	0.16
*Salmonella* Typhimurium	0.08 ± 0.01	0.02 ± 0.01	0.01
*Pseudomonas aeruginosa*	0.74 ± 0.01	1.02 ± 0.05	0.17

* *p-*values were calculated using the Student’s t-test

## Discussion

Ecological competition among microorganisms results from nutrient and antibiotic stress as well as space limitations. This competition is a powerful selection pressure that is often mediated through the synthesis and secretion of bioactive metabolites by the competing bacteria [51]. These specialised bioactive metabolites, such as bacteriocins, may have profound effects on bacterial fitness in competitive multispecies communities, but generally do not play a role in the producer bacteria’s primary metabolism. A number of studies have demonstrated the potential broad-spectrum antibacterial activity of prodigiosin. It has been proposed that prodigiosin functions as a chaotropic stressor whereby disruption of the prodigiosin-treated bacteria’s plasma membrane leads to a loss of essential bacterial components such as proteins and ions [22] as well as impeding bacterial metabolism [26]. Nonetheless, the precise mechanism(s) by which prodigiosin displays antibacterial activity, remains poorly understood. In this study, we were interested in potential antibacterial mechanisms exhibited by prodigiosin, particularly in interspecies competition. Hence, the physiological role of prodigiosin from *S*. *marcescens* was investigated. We tested the antimicrobial property of prodigiosin towards four clinical strains (MRSA, *E*. *faecalis*, *Salmonella* Typhimurium and *P*. *aeruginosa*) and two standard laboratory strains (*S*. *aureus* and *E*. *coli*). We were also interested to investigate if prodigiosin is advantageous to the survival of *S*. *marcescens* by inhibiting the production of known Gram-positive and Gram-negative bacterial virulence factors involved in microbial competition such as proteases [52] and the ability to form biofilm [53]. To obtain prodigiosin for all the assays, *S*. *marcescens* Sma 274 was cultured in PGB (pH 7) at 28°C for 96 hours [37]. Sma 274 grown under these parameters produced a total of 891.61 units/cell of prodigiosin with a final concentration of 800 μg/μL.

### Prodigiosin selectively antagonizes Gram-positive bacteria

Prodigiosin has a tripyrrole ring structure [2] and cyclic molecules generally exhibit distinct antibacterial activity compared to linear molecules [54]. Additionally, cyclic molecules are metabolically more stable and have higher cell permeability compared to their linear counterparts [55]. Prodigiosin is reported to have broad-spectrum antimicrobial activity towards both Gram-positive and Gram-negative bacteria [56]. Here, we showed that prodigiosin is more selective towards Gram-positive bacteria, an observation also noted in previous studies [9, 15]. On the other hand, prodigiosin antimicrobial activity on Gram-negative bacteria appears to be strain-dependent. Gulani et al. [9] reported that *E*. *coli* is resistant to prodigiosin as is *E*. *coli* KCTC 1116 [57]. However, we noted that our *E*. *coli* laboratory strain, *E*. *coli* OP50, is susceptible to prodigiosin, which is consistent with *E*. *coli* MG1655 susceptibility towards prodigiosin [26]. We also noted that *P*. *aeruginosa* PA14 and *Salmonella* Typhimurium SL1344 are resistant towards prodigiosin at both tested concentrations. While *P*. *aeruginosa* [9] and *P*. *aeruginosa* UFPEDA 39 [58] are also resistant to prodigiosin, *P*. *aeruginosa* MCTC 1688 [59] and *P*. *aeruginosa* KCCM 11266 [57] are susceptible to prodigiosin. Nonetheless, *Salmonella* Typhimurium resistance to prodigiosin appears to be more consistent. *Salmonella* Typhimurium SL1344 (this study), *Salmonella* Typhimurium KCTC 1926 [54] and *Salmonella* Typhimurium KCCM 40253 [57] are all resistant to prodigiosin’s antibacterial activity.

*P*. *aeruginosa* resistance towards prodigiosin could be attributed to the known production of rhamnolipids, a biosurfactant [60]. Microbial surfactants exhibit strong emulsification of hydrophobic compounds [61]. As prodigiosin is a hydrophobic compound, *P*. *aeruginosa* rhamnolipids could have emulsified prodigiosin and blocked its antimicrobial activity. Alternatively, multidrug resistance of *P*. *aeruginosa* clinical isolates is attributed to the presence of several efflux pumps such as MexAB-OprM, MexCD-OprJ, MexEF-OprN and MexXY-OprM [62]. Among these efflux pumps, the *mexCD-oprJ* operon is induced by disinfectants and cytotoxic agents [63]. Since prodigiosin was reported to have cytotoxic effects on cancer cell lines [6], it is possible that the presence of prodigiosin activated the MexCD-OprJ pump which ejected prodigiosin from *P*. *aeruginosa*. Additionally, *mexEF-oprN and mexXY-oprM* are induced by nitroaromatic antimicrobial agents [64] and aminoglycosides, [65] respectively. Under laboratory conditions, the *P*. *aeruginosa* MexAB-OprM pump confers antibiotic resistance to most β-lactams except imipenem as well as other antimicrobial agents, including chloramphenicol, tetracycline, quinolones and macrolides [66]. As MexAB-OprM also induces the production of other bacterial virulence factors such as pyocyanin, exotoxin A, proteases, exoenzyme S and rhamnolipids [67], the combinatorial effect of *P*. *aeruginosa* efflux pumps and virulence factors may have obstructed the antimicrobial effects of prodigiosin. Similarly, the resistance of *Salmonella* Typhimurium towards prodigiosin could also be due to the bacterial intrinsic multidrug resistance, including secretion of enzymes to inactivate antimicrobial drugs, decreasing cell permeability to antibiotics, modifying cellular drug targets and activating efflux pumps to remove drugs [68]. Nevertheless, the ability of efflux pumps to push out prodigiosin remains unknown and requires further investigation.

Taken together, prodigiosin is a more potent inhibitor of Gram-positive bacteria while different strains of Gram-negative bacteria display variable levels of susceptibility. While the antagonistic nature of prodigiosin towards Gram-positive bacteria is well noted [9, 15], the underlying antibacterial mechanism is still unknown. Previously, chaotropic-mediated stress of the bacterial plasma membrane was proposed as a primary mode-of-action for the antimicrobial activity of prodigiosin [22]. The more pronounced growth inhibition effect of prodigiosin on Gram-positive bacteria relative to Gram-negative bacteria, either on its own or in complex with antibiotics, may be due to the thicker and diversified peptidoglycan structure of Gram-positive bacterial membranes [69]. Although *S*. *marcescens* co-exists with other Gram-positive and Gram-negative bacteria in its natural habitat, in the presence of these bacteria under stressful conditions, prodigiosin may have a greater effect on the survival of competing Gram-positive bacteria due to the lipophilic characteristic of prodigiosin from the presence of the chain of the monopyrrole C-ring in prodigiosin (Fig 1) [24]. The thick peptidoglycan layer of Gram-positive bacteria is not an effective permeability barrier [70] and once prodigiosin overcomes the bacterial cell wall barrier, the lipophilic C-pyrrole ring preferably binds to the hydrophobic core of double-stranded DNA (dsDNA) [71]. Additionally, the bipyrrole AB-ring of prodigiosin (Fig 1) is an essential moiety group in copper-promoted dsDNA damage [72]. As reviewed by Gates [73], lipophilic plant resorcinols bind to Cu^2+^ to induce DNA cleavage in aerobic basic conditions. Similar to resorcinols, prodigiosin also binds with Cu^2+^ to form metalloprodigiosin that promotes oxidative site-directed dsDNA cleavage [74], causing cell lysis. The lysed competitor bacteria release their cytoplasmic nutrients into the surrounding milieu, which is then acquired by *S*. *marcescens* during interspecies competition [75].

The lipopolysaccharide structure on the outer polysaccharide membrane of Gram-negative bacteria is impermeable to lipophilic solutes [70]. Hence, Gram-negative bacteria are less penetrable by the lipophilic prodigiosin compared to Gram-positive bacteria. Furthermore, the similarity in bacterial cell wall structure between *S*. *marcescens* and other Gram-negative bacteria may have rendered prodigiosin less effective towards Gram-negative bacteria. Although Gram-negative bacteria are generally more resistant towards prodigiosin, a pleiotropic effect was observed in some Gram-negative bacteria treated with prodigiosin. An impairment of several key cellular functions such as cell division and respiration, RNA and protein synthesis as well as the integrity of outer membrane was observed in prodigiosin-treated *E*. *coli*. However, DNA is not the main target of prodigiosin in *E*. *coli* [26].

A limitation of our study is the use of crude prodigiosin extract which resulted in an MIC generally higher than that previously reported for purified prodigiosin. For example, the MIC of prodigiosin purified from *Vibrio ruber* DSM14379 towards *E*. *coli* was 103.4 ± 6.3 μg/mL [25], purified prodigiosin from *Serratia* sp. PDGS 120915 has an MIC value of 32 μg/mL against MRSA [76] and purified prodigiosin from *S*. *marcescens* UFPEDA 398 had low MIC values (1.0–4.0 μg/mL) against eighteen oxacillin-resistant *S*. *aureus* (ORSA) clinical strains [58]. Although the absorption spectra, HPLC and LC-MS/MS analyses (Figs 2 and 3) showed that prodigiosin is a predominant compound in the crude extract, nevertheless, the higher MIC value may likely be a result of minor impurities within the crude prodigiosin extract which could have impeded the action of prodigiosin or its interaction with the bacterial membrane, thus, requiring higher prodigiosin concentrations to inhibit bacterial growth. While the findings from this study propose that the crude prodigiosin extract targets Gram-positive bacteria cell wall leading to cell lysis, we also asked if prodigiosin physiologically modulates other known bacterial virulence determinants.

### Prodigiosin inhibits bacterial virulence factors involved in interspecies competition

Bacterial virulence factors reported to be important in microbial dominance include production of proteases [52] and formation of biofilm [53]. We also investigated the effect of prodigiosin towards haemolysin, an exotoxin secreted by a number of clinical pathogens that lyses red blood cells [77, 78] to enable iron acquisition by the infecting bacteria [79]. β-haemolysin secreted by *S*. *aureus* also promotes skin colonisation by damaging keratinocytes [80]. In the presence of prodigiosin, haemolysis of red blood cells by the selected pathogenic bacteria was not inhibited. This suggests that prodigiosin may typically respond to factors produced during interspecies competition but has no affinity for haemolysin that is only secreted by pathogenic bacteria when present in the infected host [81].

Extracellular protease is an important virulence factor secreted by pathogens not only during host infection but also to degrade soil proteins into amino acids and peptides for nutritional benefits [52] when competing against other microbes. Similarly, when faced with a competitive natural environment, pathogenic bacteria are known to secrete proteases that are toxic to other bacterial species. For example, the *P*. *aeruginosa LasR* mutant secretes many virulence factors including proteases that are toxic to other microbes in addition to eukaryotic cells [82]. *Pseudomonas fluorescens* CHA0 secretes extracellular proteases to suppress the nematode *Meloidogyne incognita* [83]. Guided by these reports, we asked if *S*. *marcescens* secretes prodigiosin to inhibit proteases secreted by other environmental bacteria in order to survive in competitive multispecies niches such as soil and surface water. We treated the different bacteria cultures with prodigiosin and monitored proteolysis of casein present in skim milk. Prodigiosin significantly inhibited proteolytic activity of proteases secreted by MRSA, *E*. *faecalis*, *E*. *coli* and *P*. *aeruginosa*. As we had noted above, prodigiosin inhibits the growth of MRSA, *S*. *aureus*, *E*. *facealis* and *E*. *coli* used in the current study, hence, the reduction in proteolytic activity of prodigiosin-treated bacteria may be due to lower bacterial cell density and therefore, reduced protease secretion as compared to the untreated cultures. On the other hand, the prodigiosin anti-proteolytic effect could directly target proteases. This is likely true for *P*. *aeruginosa* whereby prodigiosin failed to inhibit growth (Fig 4; Table 1) but a notable reduction in the size of the proteolytic zone (Fig 6) was observed for prodigiosin-treated bacteria. Previously, protease activity of *Aedes aegypti* larvae treated with prodigiosin was reduced by 36% [84]. As small molecules can interact with biological macromolecules [26], prodigiosin could bind to the allosteric site of the proteases, inducing conformational changes which results in the loss of proteolytic activity.

Planktonic bacteria attach to biotic and abiotic surfaces to associate with each other to form biofilm, an exopolysaccharide matrix [85], in response to factors such as specific and non-specific cellular recognition, nutrient depletion signals or harsh conditions [86]. In addition, a major inducer in biofilm formation in nature is competition with other microbial species and strains [53]. Only limited information is currently known about the effect of prodigiosin on bacterial biofilm formation [35, 87]. To assess the possibility that *S*. *marcescens* secretes prodigiosin to inhibit biofilm formation and enable dispersal of competitor bacteria, we measured biofilm formation in prodigiosin-treated and untreated bacteria. Biofilm formation by prodigiosin-treated *E*. *faecalis*, *Salmonella* Typhimurium and *E*. *coli* was significantly reduced compared to untreated bacteria (Table 4). The reduction in biofilm formation for prodigiosin-treated *E*. *faecalis* and *E*. *coli* correlates with the growth inhibition noted above, reducing the number of planktonic cells adhering to the surface of the well and concomitant reduction in aggregation and biofilm formation. On the other hand, the growth of *Salmonella* Typhimurium is unaffected by prodigiosin at up to 500 μg/μL (Fig 4; Table 1) suggesting that prodigiosin inhibits *Salmonella* Typhimurium biofilm formation by negatively regulating the pathways involved in biofilm formation or alternatively, prodigiosin disrupts the bacterial biofilm structure.

Interestingly, both *S*. *aureus* and *P*. *aeruginosa* treated with prodigiosin showed increased biofilm formation, with significant biofilm formation observed in *S*. *aureus* cultures. MRSA treated with prodigiosin showed no changes in biofilm formation when compared to the untreated culture (Table 4). It is possible that MRSA, being an antibiotic resistant clinical strain, is similarly able to resist the effect of prodigiosin. Our results indicated that MRSA is sensitive towards prodigiosin but at a higher prodigiosin MIC value (>10 μg/μL). The higher amount of prodigiosin required to inhibit MRSA growth could be due to the MRSA biofilm formation. This suggests that the biofilm of MRSA is an effective protective barrier against prodigiosin.

Reduced biofilm formation was previously noted for *P*. *aeruginosa* PA14 in the presence of prodigiosin-Cu^2+^ complex which cleaved dsDNA [74]. Extracellular DNAs (eDNAs) are essential components of biofilm [88], thus, removal of eDNAs by prodigiosin-Cu^2+^ disrupted biofilm formation [35]. In our study, prodigiosin without the addition of Cu^2+^ led to an increase in *P*. *aeruginosa* PA14 biofilm formation. It is possible that the presence of prodigiosin presented increased stress on both *S*. *aureus* and *P*. *aeruginosa* cultures and therefore, the planktonic cells quickly aggregated to form biofilm and deter the antimicrobial effect of prodigiosin. Alternatively, prodigiosin could also stimulate biofilm formation in both *S*. *aureus* and *P*. *aeruginosa* by inducing biofilm-related genes. Bagge et al. [89] noted an increase in *P*. *aeruginosa* biofilm in the presence of imipenem due to the overexpression of alginate, an important polysaccharide component in *P*. *aeruginosa* biofilm. Similarly, biofilm formation was also induced in *P*. *aeruginosa* treated with aminoglycosides [90]. Previously, it was reported that biofilm formation of *B*. *subtilis* was induced when the culture was treated with small antimicrobial molecules that promote K^+^ ion leakage from bacterial cells [91]. An increase in biofilm formation was also noted for *S*. *aureus* treated with β-lactam drugs and this effect was associated with autolysin-dependent release of eDNAs by Gram-positive pathogens when treated with antibiotics [92]. Hence, induction of biofilm formation in the presence of prodigiosin could be a strategy employed by *P*. *aeruginosa* and *S*. *aureus* to outcompete prodigiosin-producing hosts in a given environment such as in soil and surface water. Taken together, we suggest that when pigmented *S*. *marcescens* co-exists in the same environmental niche as bacteria that aim to gain a survival advantage by forming biofilm, *S*. *marcescens* produces prodigiosin to disperse the biofilm and subsequently secretes other virulence factors to kill the competitor bacteria.

### *S*. *marcescens* secretes prodigiosin to survive a competitive multispecies environment

Our results demonstrated that the microbial growth and virulence factors of clinical pathogens are generally more resistant to prodigiosin compared to laboratory strains. Our results also mirror the resistance of clinical strains such as *E*. *coli*, *P*. *aeruginosa* [9], *Salmonella* Typhimurium KCTC 1926 [54], *E*. *coli* KCTC1116, *Salmonella* Typhimurium KCCM 40253 [57] and *P*. *aeruginosa* UFPEDA 39 [58] towards prodigiosin. However, MRSA and *E*. *faecalis* are more susceptible to prodigiosin compared to *Salmonella* Typhimurium and *P*. *aeruginosa*. As discussed above, the susceptibility of Gram-positive bacteria towards prodigiosin could be attributed to the structural differences in bacterial cell wall. Nevertheless, it is also possible that prodigiosin inhibits MRSA by its memory of environmental *S*. *aureus* co-habiting in the same soil environment and explains why prodigiosin is equally inhibitory towards MRSA and *S*. *aureus*. In a previous report, eighteen ORSA clinical isolates were also susceptible to prodigiosin [58], further supporting that prodigiosin antagonises both MRSA and ORSA through *S*. *aureus* recognition. Although *E*. *faecalis* is a mammalian intestinal commensal [93], this pathogen is commonly found in the environment due to fecal contamination [94] and thus, prodigiosin could also have evolved to target *E*. *faecalis*. Moreover, according to Mahlen [95], environmental *S*. *marcescens* isolates are normally pigmented; supporting our hypothesis that prodigiosin is only secreted in the environment in response to interspecies competition. Collectively, prodigiosin inhibits bacterial growth and biofilm formation as well as deregulates bacterial protease activity but clinical isolates are generally more prodigiosin-resistant, further supporting the physiological role of prodigiosin when in competition with other microbes.

## Conclusion

In summary, secretion of the antibacterial prodigiosin is most likely a strategy employed by *S*. *marcescens* during environmental interspecies competition. Prodigiosin, as a potent antibacterial agent, inhibited bacterial growth with higher selectivity towards Gram-positive bacteria and is antagonistic towards the six tested pathogens using different mechanisms. These mechanisms may include higher prodigiosin cell permeability through interaction with the peptidoglycan structure of Gram-positive bacteria, disruption of bacterial protease secretion or proteolytic activity as well as reduction in biofilm formation. Results from this study strongly suggest that *S*. *marcescens* secretes prodigiosin to compete against other bacteria and gain a survival advantage as well as to establish itself in its natural ecological niche such as soil and surface water.

## Supporting information

S1 Appendix (PDF)
