# Supplementary material for: Prodigiosin inhibits bacterial growth and virulence factors as a potential physiological response to interspecies competition
Source: PLoS One. 2021 Jun 23;16(6):e0253445. doi: 10.1371/journal.pone.0253445 (PMC8221495; doi:10.1371/journal.pone.0253445)
Supplement: S1 Appendix — (PDF) [file pone.0253445.s001.pdf]

S1 Appendix

S1 Table. Inhibition zones of each bacterial replicate treated with 250 µg/µL and 500 µg/µL of prodigiosin after 24 hours.

**Bacteria**	**Replicates**	**Diameter of inhibition zone (mm)**				
		**Positive control**	**Negative control**		**Prodigiosin**	
		**Chloramphenicol (35 µg/µL)**	**EtOH**	**MeOH**	**250 µg/µL**	**500 µg/µL**
Methicillin-resistant *Staphylococcus aureus*	1	23	0	0	20	21
	2	23	0	0	19	21
	3	22	0	0	20	21
Staphylococcus aureus	1	30	0	0	20	21
	2	25	0	0	20	22
	3	25	0	0	20	22
Enterococcus faecalis	1	20	0	0	18	20
	2	18	0	0	21	20
	3	20	0	0	20	21
Escherichia coli	1	28	0	0	21	26
	2	31	0	0	23	27
	3	38	0	0	22	24
Salmonella Typhimurium	1	27	0	0	0	0
	2	29	0	0	0	0
	3	29	0	0	0	0
Pseudomonas aeruginosa	1	26	0	0	0	0
	2	25	0	0	0	0
	3	0	0	0	0	0

S2 Table. Area of proteolytic zones following prodigiosin treatment on each of the bacterial replicate after 24 hours.

**Bacteria**	**Replicates**	**Area of inhibition zone (mm^2)**		
		**MeOH**	**- Prodigiosin**	**+ Prodigiosin**
Methicillin-resistant *Staphylococcus aureus*	1	24	30	22
	2	22	28	23
	3	25	26	23
Staphylococcus aureus	1	24	35	24
	2	23	28	25
	3	24	25	25
Enterococcus faecalis	1	24	25	23
	2	25	24	24
	3	24	25	23
Escherichia coli	1	11	13	11
	2	10	12	11
	3	10	13	10
Salmonella Typhimurium	1	0	0	0
	2	0	0	0
	3	0	0	0
Pseudomonas aeruginosa	1	18	21	21
	2	20	22	21
	3	21	22	20

S3 Table. Formation of biofilm by each prodigiosin-treated and -untreated bacterial replicate after 48 hours incubation.

**Bacteria**	**Replicates**	**Absorbance readings at 570 nm**	
		**- Prodigiosin**	**+ Prodigiosin**
Methicillin-resistant *Staphylococcus aureus*	1	0.15	0.18
	2	0.16	0.20
	3	0.28	0.23
Staphylococcus aureus	1	0.26	0.65
	2	0.36	0.91
	3	0.21	0.62
Enterococcus faecalis	1	0.75	0.40
	2	0.71	0.44
	3	0.89	0.51
Escherichia coli	1	0.09	0.06
	2	0.10	0.10
	3	0.17	0.04
Salmonella Typhimurium	1	0.06	0.02
	2	0.07	0.03
	3	0.10	0.01
Pseudomonas aeruginosa	1	0.34	0.95
	2	0.94	1.11
	3	0.94	1.00
